# 

**Published:** 1919-11-29

**Authors:** 


					Cost of Paying Patients at Johannesburg.
The Johannesburg General Hospital is incurring a loss
?on the great majority of its paying patients, owing,
seemingly, to the.high cost of living. A return was laid
before the Hospital Board at its meeting in September.
Gifts and Bequests.
The Kin:; lias contributed ?100 towards improvements
and extensions at the Norfolk and Norwich Hospital.
Sir Andrew McDonald, Lord Provost of Edinburgh, has
left ?1,000 each to the Edinburgh Royal Infirmary and to
the Royal Edinburgh Hospital for Incurables (I.ongmoro
Hospital), and ?500 each to the Royal Hospital for Sick
Children, Edinburgh, and the Scottish Blinded Soldiers'
and Sailors' Rest.
Mr. John Whelan, of Dublin, has bequeathed ?50 each
to tho Jervis Street Hospital, Dublin, and the Mater Miseri-
cordirc Hospital, and ?20 to tho Hospico for the Dying,
"Harold's Cross, Dublin.
The Queen has sent a quantity of beautiful silks and
brocades to the Royal Hospital and Home, for Incurables,
Putney, for the use of the patients in making articles foi
tho annual Salo of Work.
Subscribed by members of a Cardiff Jewish community,
n chequ:? for 1,000 guineas has been presented to King
Edward VII. Hospital.
Mb. J. J. Morrish, of Oxshott, Surrey, has kft ?500 to
Cobham Cottage Hospital.
The Prince of Wales has given ?20 to the Moffat Mater-
nity and Child Welfare Centre connected with tho Brixton
Independent Church.
It is expected that the Miller General Hospital, Green-
wich, will receivo ?800 as the result of a bazaar for a
children's ward.
At the annual Pound Day of the Surgical Ilomo for Boys,
Banstead, 320 lb. in kind and ?19 10s. in money were
received.
Large charitable bequests are made by the will of Mr.
James Morton, managing director of W. Hill and Son, Ltd.,
confectioners. ?39,COO is devoted to hospitals, including the
following bequests: ?1,000 each to the Belgrave Hospital.
Victoria Hospital, Queen's Hospital, and Great. Ormond
Street, Hospital (all. for children), and tho National Ortho-
paedic Hospital.
Mrs. Marie Wedley, of Park Lane, W., left ?17,COO and
land and buildings at Benworthy to the Win?ford Cottage
Hospital, founded by her in memory of her late husband
and nephew. Tho West Lortdon, Hospital and the Dread-
nought Hospital will benefit under tho will by the sale of a.
collection of tapestry and silver.
The Lord Mayor has received ?75,000, the proceeds of
" France's Day " collection in Great Britain and Canada.
Merthyr Tydfil General Hospital will benefit by a re-
versionary legacy of ?1,935 New Zealand Bond-, 1: ft by
Mr. Franklin Dixon, of Newport (Mon.).
November 29, 1919. THE HOSPITAL 199
The Payment of Poor Law
Chaplains.
The Halifax Guardians have decided to pay their in-
firmary chaplains. The neighbouring Union of Leeds has
paid its chaplains since 1844. In this instance the Church
of England chaplain receives ?175 per annum and bonus,
a Roman Catholic instructor ?100 per annum and bonus,
and the superintendent of the City Mission ?50 and bonus.
Reflection has convinced all but a few fanatics that no
clergyman can be expected to visit an institution, perhaps
almost daily, minister to the sick, and hold services on
Sundays, without a reasonable fee for this additional
work.
Harefield Park Sanatorium.
The Health Ministry has agreed to the scheme of the
Middlesex County Council for the purchase of Harefield
Park and the huts there for a sanatorium, and the
Treasury has agreed to make a capital grant of three-
fifths of the total capitkl expenditure, subject t-o a maxi-
mum grant of ?180 per bed provided. The cost of the
sanatorium is expected to be ?64,OCO.
New Buildings Contemplated.
IIartshorne.?A Conference will shortly take place to
consider the advisability of providing a cottage hospital
for the Woodvilie and IIartshorne district. Mr. Goodnead?
of Swadlincote, has offered ?1,000 towards the cost.
Seatbuen.?The Durham County Council has asked for
plans for the extension of tlio administrative block of the-
hospital at an estimated cost of ?1,010.
Spalding!.?At a meeting of the County Council a proposal
to purchase an estate in Lincolnshire for conversion into a
sanatorium was approved.
Swansea.?A ferro concrete structure has been suggested
for the new isolation hospital.
Wallasey.?Funds are being raised for extensions to the
Victoria Central Hospital; estimated cost ?20,000.
Whickham.?The Urban District Council has accepted an.
offer to purchase the Rectory and grounds, comprising nine
acres, for ?4,000 for conversion into a cottage hospital and
a public park.
Whitley.?A new isolation hospital, to cost ?4,000, is
under contemplation by the Urban District Council.
Wilmslow.?It has been decided to make an effort to raise
?10,000 for a cottage hospital.
Wimbledon.?As a war memorial it is proposed to dsvoto
?55,000 to a children's hospital.
Worthing.?At the last meeting of the West Sussex County
Council the revised estimate of ?4,500 was approved for
the provision of tuberculosis hospital accommodation at
Swandean, Worthing.
The Irish Nurses' Union.
At the usual weekly meeting of the Committee of
the Irish1 Nurses' Union, held at 29 South Anne Street.
Dublin, on the 4th instant, the Secretary reported on
the progress of the Union's claim for higher salaries for
district midwives. In several districts the guardians
had offered increases ranging up to ?20 per annum.
Dublin Guardians, on the Union's application, were seek-
ing Local Government Board sanction for increases
averaging about ?50 for the hospital staff, and the Union
was still negotiating on this and other improvements-
Other matters dealt with included State registration, meet-
ings in Wat-erford and Kilkenny, Dublin Corporation, ete.
?" Commtmicated.'"
A Leicester Stockbroker's Generosity*
The report of the meeting of the Leicester and County
Saturday Hospital Society (which appeared in The Hos-
pital of July 26) announced that it was hoped to establish
a Children's Convalescent Home for the children of mem-
bers, and thus complete the whole circuit of convalescent
treatment. Mr. Arthur Wheeler, a local resident, has
generously given to the Society a large house standing
in grounds of about two acres, which recently was pur-
chased by him for presentation to the Society. Th'
Home has the great -advantage of being situated near
to the Society's Home for Women at SwithLand. Th-?
gift will doubtless be thankfully accepted, and the an-
nouncement of it shows the ready willingness to promote?
the well-being of the community.
-300 1 HE. HOSPITAL N OVEMBER '29, 1919.
Matrons' and Sisters'
Appointments,
Workhouse Infirmary, Lincoln.?Miss M. E. Slant,field
lias beon appointed superintendent nurse. She was trained
at Leeds Township Infirmary, and has since been sister at
the Southern Fever Hospital, Dartford, Kent; Savernaeke
Hospital, Marlborough, and in Q.A.I. M.N.S.R. Miss
Stansfield holds the certificate of the C.M.B.
Poor-Law Infirmary, Newcastle-on-Tyne.?Miss Louisa
Smith and Miss Ethel Madden have been appointed waril
sisters. Miss Smith was trained at Bromley Union In-
firmary, where she became ward sister. Miss Madden was
trained at Tynemouth Union Infirmary, and has since done
private nursing in Sunderland.
County Tuberculosis Hospital, Hertfordshire County
Council, Ware, Herts.?Miss M. Sutton has been appointed
matron. She was trained at the Royal South Herts Hos-
pital, Southampton, and has held the appointments of
sister and deputy-matron of the Jones' Memorial Home,
Lanes; health visitor for Nottingham, and Notts Association
for the Prevention of Consumption; acting sister in
Q.A.I.M.N.S.R. on active service 111 France, and sister-in-
charge of an ambulance barge.
Hospital for Children, Leaf owe, Cheshire.?Miss R.
Todd has been appointed ward sister. She was trained at
the London Hospital, where she became holiday sister, and
has also been sister-in-charge Newland Corner Auxiliary
Hospital, Guildford.
Minkhead and Dunster Hospital, Dunster, Somerset.?
Miss N. Courteen has been appointed sister. She was
trained at the Bristol Royal lnhrmary, arid has served in
France under the French Ked Cross. Sue has the certificate
of the C.M.B.
Bank Hall Maternity and Children's Hospital, Burnley.
?Miss D. Widdar has been appointed senior sister, bne
was trained at. the Cumberland Innrmary, Uariisle, where
she was later ward, night sister, and assistant matron. She
has done private nursing, and holds the C.iU.B. certificate,
for which she trained at the Bristol Royal Infirmary.
Birmingham and Midland Eye Hospital.?Miss M. E.
Thompson and Miss E. Waters have been appointed sisters.
Miss Tnompson was trained at the Koyal Innrmary, Shef-
field, and has since been sister, T.F.N.S., at the 3rd .Northern
General Hospital, Sheffield. Miss Waters was trained at
the North Kiding Infirmary, Middlesbrough, and has been
four years in. Q.A.I.M.N.S.R.
Prescot InfIRmary, Lancashire.?Miss Dorothy Brown,
Miss W. E. Moss, and Miss A. Houghton have been pro-
moted ward sisters at the above institution, where they
receive d their training.
Forthcoming Event.
Royal Hospital and Home for Incurables.?Founders'
Day Celebration, Tuesday, December 2, 1919, 7 p.m.
Hospital Pharmacist Bccomcs Borough
Councillor.
Miss Gilliatt, pharmacist and dispenser at the Western
Fever Hospital of the Metropolitan Asylums Boarel, has
been elected a member of the Fulham Borough Council as
a representative of the Labour party.
Miss Gilliatt is well known in pharmaceutical circles,
being President of the Women Pharmacists' Association
(London), and also a member of the Council of the Public
Pharmacists' Association, also representing institutional
pharmacists on the Executive of the amalgamated Society.
We think this is the first instance of a hospital pharmacist
undertaking- the duties of a borough councillor, and ha.vo
no doubt that Miss Gilliatt's professional knowledge will
prove valuable?more especially in regard to public-health
work?to the electors whom she represents.
The College of Nursing
(Limited by Guarantee).
LIVERPOOL CENTRE.
The Club which has been organised for this Centre will
be open to members on Monday, Decembor 1. The Club,
which is in a very good central position at No. 38 Church
Street, has the further advantage of a lift.
EDINBURGH CENTRE.
The Annual Meeting of the Edinburgh Centre was held
in the Goold Hall, 5 St. Andrew Square, on Wednesday,
November 19. Miss Gill, R.R.C., presided. Invitations
had been extended to all College members, whether belong-
ing to the Centre or not, and the attendance was excellent.
Participation in the business proceedings was confined to
Centre members only. The Hon. Secretary read the Report
for the year, the adoption of which was moved by Miss
Lamb, and seconded by Miss J. Macgregor. The following
oflieo bearers .and members of the Executive for the coming
year (proposed by Miss Hyde, Q.V.J.N.I., seconded by MisS
Wardrop) were elected unanimously by the meeting: Miss
Gill, President j Miss Cumming, Chairman of the Executive ;
Miss Turnbull and Miss Cathoart, Joint Hon. Treasurers
and Secretaries. The eight retiring members of the Execu-
tive were re-elected, with the addition of four new members
namely, Miss Ritehie, Miss Calderhead, Mrs. Bowie, and
Miss Milligan, R.R.C.
Reference was made to the winter programme of lectures
and social events, details of which will be published in due
course. Miss Cumming drew the attention of the meeting
to the Bill for State Registration of Nurses which had just
passed its second reading in the House of Commons.
Afterwards Mrs. Blaikie, M.D., gave an interesting and
inspiring address on " Trades Unions in Relation to the
Nursing Profession."
Forty new members joined the Centre. At the conclusion
of the meeting tea. was served.
OXFORD: NEW LOCAL CENTRE FORMED.
(Hon. Secretary: Miss Hayes, 108 Woodstock Road, Oxford.)
At an enthusiastic meeting held on Friday, November 21,
at the Radclifie Infirmary, it was unanimously resolved to
form a local Centre for Oxford, and the necessary steps were
taken to put this resolution into action. The activity and
healthy growth of the older Centres will undoubtedly spur
Oxford to prove itself worthy of the lecture and educa- .
tional facilities which the University city possesses, which
should make the progress of this Centre and its rapid de-
velopment an easy matter.
King Edward Memorial Home, Edinburgh-
The Nurses of the Western Infirmary held a sale of
work on Saturday, November 22, on behalf of the " King
Edward Memorial Home" for Nurses in Scotland. As a
result of the sale the Matron was able to forward to the
Honorary Secretary of the Home the sum of ?420 from
the nursing staff. This splendid result should '_??
memorable for all time.
" MABELA."
AN INVALID FOOD.
Those who suffer from any form of indigestion and
are in search of a food which they can rely upon as
having all the nutritive values, yet amenable to the de-
bilitated digestion, should try " Mabela." "Mabela" has
been highly recommended by those using it, and is a
farinaceous food of the malt" variety prepared from the
product of a South African grain. It is palatable, easily
prepared, and has been proved to be readily assimilated
where similar foods have been rejected; an undoubted
restorative in mental and physical exhaustion, and of
equal value for growing children. " Mabela" is prepared
by Hind Bros, and Co., Durban, Natal, and can be
obtained from the British agents, Francis Newbery and
Sons, Ltd., London, in sealed tins at a very nominal
cost.

				

